# Development of Multi-Vessel Coronary No-Reflow Following Elective Percutaneous Intervention in One Vessel

**DOI:** 10.7759/cureus.48182

**Published:** 2023-11-02

**Authors:** Syed W Hussain, Eddison Ramsaran

**Affiliations:** 1 Department of Cardiology, Saint Vincent Hospital/UMass Chan Medical School, Worcester, USA; 2 Department of Cardiovascular Medicine, Saint Vincent Hospital, UMass Chan Medical School, Worcester, USA

**Keywords:** coronary vessel disease, culprit lesion, elective angioplasty, primary percutaneous coronary intervention (pci), no-reflow

## Abstract

Percutaneous coronary intervention (PCI) is a frequently performed procedure that can have minor or major complications. One of the more serious complications of PCI is the development of coronary no-reflow. No-reflow signifies reduced or absent coronary flow in the distal coronary circulation in the absence of flow-limiting lesions. We present a case of a middle-aged man who presented as an outpatient for elective coronary angiography due to angina pectoris and a high-risk exercise stress test. Coronary angiography demonstrated significant single-vessel disease with lesions in the proximal and mid-segments of the left anterior descending (LAD) coronary artery. Successful placement of drug-eluting stents in the LAD was followed by a severe drop in blood pressure, worsening chest pain, and ST elevation on telemetry. Immediate angiography showed the development of no-reflow in both the LAD and left circumflex coronary arteries. Ionotropic and intravenous anti-platelet agents were administered with simultaneous placement of an intra-aortic balloon pump, restoring normal flow in both arteries. No-reflow occurs most commonly following PCI in certain lesion subsets, and it is usually seen only in the vessel in which the PCI was performed (culprit vessel). It is important to realize that this phenomenon can occur in other circumstances since immediate recognition and treatment can be lifesaving.

## Introduction

In the United States, there are approximately 900,000 percutaneous coronary interventions (PCIs) performed each year [1]. Complications are uncommon but can be severe. One of these is the occurrence of no-reflow, which is the impediment of blood flow caused by obstruction of the distal coronary microvasculature [2]. It is well known that this is most often seen following PCI of specific lesion subsets and is then usually only seen in the culprit’s vessel [2]. We present a case in which, following PCI of a stable lesion in an outpatient setting, there was the development of multi-vessel no-reflow, causing hemodynamic collapse. We elaborate on the possible mechanism for this occurrence.

## Case presentation

A 60-year-old male with a history of pre-diabetes, hyperlipidemia, and subclinical hypothyroidism presented to the cardiology clinic due to concerns about coronary artery disease. He had a significant family history of premature coronary artery disease, and he had been complaining of mild chest pressure. An exercise stress test was abnormal, with significant ST depression seen in V5, V6, and AVF. He was referred for coronary angiography. On examination, his blood pressure was 154/74 mmHg, his heart rate was 69 bpm, and he was afebrile with a saturation of 98% on room air. Heart examination demonstrated normal first and second heart sounds with no murmurs, and respiratory examination demonstrated normal breath sounds. He has no peripheral edema. Cardiac catheterization was performed via the right radial artery, and a 6 French Tiger 4.0 catheter was used for the angiography. This demonstrated no disease involving the left main, left circumflex, or right coronary arteries, but there was a moderate proximal and a long segment of 90% stenosis involving the mid-left anterior descending (LAD) artery (Video 1).

**Video 1:** Right anterior oblique cranial view showing significant stenosis in the left anterior descending artery prior to intervention.

A decision was made to intervene. A 6 French EBU (Extra Backup Catheter) 3.5 guide catheter was used to engage the left main coronary artery. The lesion in the mid-left anterior descending coronary artery was treated with pre-dilation using a semi-compliant balloon (2.0 mm × 8 mm) at 10 Atm. The stent could not be delivered due to the proximal disease, so another semi-compliant balloon (3.0 mm × 20 mm) was used. This was followed by a non-compliant balloon to further pre-dilate the lesion. Eventually, a Resolute Onyx stent (2.5 mm × 15 mm) was deployed in the mid-segment of the vessel. Due to diffuse disease proximally, two additional Resolute Onyx stents (sizes: 3.0 mm × 18 mm and 2.5 mm × 15 mm) were placed. Immediately after the successful deployment of the stent, the patient developed severe hypotension with no-reflow (thrombolysis in myocardial infarction, TIMI, flow 0) in the left anterior descending artery and left circumflex artery (Video 2). The electrocardiogram showed ST elevation in the inferior and anterior leads.

**Video 2:** Right anterior oblique cranial view with no-reflow in the left circumflex and left anterior descending arteries.

Fluoroscopy demonstrated minimal cardiac contractility. Activated clotting time at this time was high (above 350). A bolus of neo-synephrine was given, and a norepinephrine infusion started. An intra-aortic balloon pump (size: 8F 50 cc) was rapidly inserted via the right femoral artery. The blood pressure stabilized immediately with a resolution of ST elevation. The EBU 3.5 Guide catheter was replaced by a 6-French EBU 4.0 Guide for better guide support. The coronary angiogram showed improved TIMI 3 flow in the left anterior and left circumflex coronary arteries. Subsequently, the more proximal lesions were treated by using Resolute Onyx stents (sizes: 3 mm × 18 mm and 3 mm × 12 mm). The final angiogram showed excellent angiographic results with TIMI 3 flow (Video 3).

**Video 3:** Right anterior oblique cranial view showing restoration of flow in the left circumflex and left anterior descending arteries.

An infusion of eptifibatide was started. Due to normalized hemodynamics, the intra-aortic balloon pump was removed immediately after the procedure. An echocardiogram in the recovery room demonstrated a normal ejection fraction with no pericardial effusion. The patient was discharged home the next day with dual antiplatelet agents.

## Discussion

In the catheterization laboratory, after successful percutaneous coronary intervention of a coronary artery, reduced or no flow in that artery is known as no-reflow [2,3]. In the literature, it has been described as occurring in 0.6% to 5% of PCIs [4]. It is seen more often in acute myocardial infarction, high-risk graft cases, and during calcium modification procedures [4-6]. Thrombotic lesions are more likely to lead to no-reflow than non-thrombotic lesions (50% of PCI cases) [4,7].

The pathogenesis of no-reflow is multifactorial and includes endothelial injury with occlusion of the micro-vessels due to distal embolism and external mechanical compression secondary to edema from infarcted myocardium [8,9]. There is also reperfusion injury with disruption of endothelial cells, generation of oxygen-free radicals, and neutrophil plugging [10,11].

However, this does not explain why there can be no-reflow in the non-culprit artery. Interestingly, a publication in 1999 showed that in the presence of myocardial infarction, following PCI flow can be compromised not only in the culprit artery but also in the non-culprit artery by up to 45% [1].

As a result, another mechanism has been proposed. This involves the development of diffuse vasoconstriction and necrosis caused by the release of neuro-humoral factors into the shared microvasculature of both the culprit and non-culprit vessels, resulting in multi-vessel no-reflow [4,12]. This, we believe, is what occurred in our case.

However, before a diagnosis of diffuse no-reflow is made, it is important to exclude other potential causes that impede blood flow. Such as an extensive proximal coronary dissection, diffuse air embolism, deep engagement of the guiding catheter, and extensive proximal thromboembolism. When these factors are excluded, one also must consider whether it is possible that impediments from non-culprit artery filling could have developed due to the hemodynamic perturbation or drop in perfusion pressure caused by the slow flow phenomenon in the culprit artery. However, postmortem selective coronary angiography studies have demonstrated that the rate of ante-grade filling is well preserved [3].

Management options for no-reflow could range from non-pharmacologic techniques utilizing thrombus aspiration and distal embolization protection devices in the setting of thrombotic occlusion [13-15]. Glycoprotein IIb/IIIa inhibitors can be utilized in the setting of acute myocardial infarction, secondary to their positive impact on the reduction of infarct size. Vasodilators, including nicardipine and verapamil, have been shown to be effective in improving distal coronary flow [16]. Although nitroprusside did not show any mortality benefit in prior studies, it can be used along with adenosine to improve distal flow [5]. Intra-coronary epinephrine has also been shown to be helpful in no-reflow [17].

## Conclusions

No reflow has short- and long-term consequences if not treated promptly. These include ischemia, infarction, cardiogenic shock, and even death. The phenomenon can be present in both emergent and elective PCI cases and, more importantly, can occur in non-culprit vessels. Hence, the practitioner must be aware of this since early recognition and prompt action can be lifesaving.
